# Cycle length of nonsustanied ventricular tachycardias among ICD patients: implications on subsequent appropriate therapies

**DOI:** 10.1186/s12872-021-02087-2

**Published:** 2021-05-31

**Authors:** Javier Jiménez-Candil, Olga Duran, Armando Oterino, Jendri Pérez, Juan Carlos Castro, Jesús Hernández, José Moríñigo, Manuel Sánchez García, Pedro L. Sánchez

**Affiliations:** Arrhythmias Unit, Cardiology Department, IBSAL-University Hospital, CIVER-CV, Universidad de Salamanca, Paseo de San Vicente, 58-182, 37007 Salamanca, Spain

**Keywords:** Implantable cardioverter-defibrillator, Ventricular tachycardia, Appropriate therapy

## Abstract

**Background:**

ICD patients with episodes of nonsustained ventricular tachycardias (NSVT) are at risk of appropriate therapies. However, the relationship between the cycle length (CL) of such NSVTs and the subsequent incidence of appropriate interventions is unknown.

**Methods:**

416 ICD patients with LVEF < 45% were studied. ICD programming was standardized. NSVT was defined as any VT of 5 or more beats at ≥ 150 bpm occurred in the first 6 months after implantation that terminated spontaneously and was not preceded by any appropriate therapy. The mean follow-up was 41 ± 27 months.

**Results:**

We analyzed 2201 NSVTs (mean CL = 323 ms) that occurred in 250 patients; 111 of such episodes were fast (CL ≤ 300 ms). Secondary prevention (HR = 1.7; *p* < 0.001), number of NSVT episodes (HR = 1.05; 95% CI 1.04–1.07; *p* < 0.001) and beta-blocker treatment (HR = 0.7; *p* = 0.04) were independent predictors of appropriate interventions; however, the mean CL of NSVTs was not (*p* = 0.6). There was a correlation between the mean CL of NSVTs and the CL of the first monomorphic VT: r = 0.88; *p* < 0.001. This correlation was especially robust in individuals with > 5 NSVTs (r = 0.97; *p* < 0.001), with an agreement between both values greater than 95%. Patients with any fast NSVT experienced a higher incidence of VF episodes (26%) compared to those without NVSTs (3%) or with only slow NSVTs (7%); *p* < 0.001.

**Conclusions:**

Unlike the burden, the CL of NSVTs is not a predictor of subsequent appropriate interventions. However, there is a close relationship between the CL of NSVTs and that of arrhythmias that will later lead to appropriate therapies.

## Introduction

Episodes of nonsustained ventricular tachycardia (NSVT) are frequently observed in subjects with left ventricular systolic dysfunction and heart failure [1, 2]. Among ICD patients, the prevalence of such episodes ranges from 20 to 60% [3–5], depending on the duration of monitoring, detection criteria and clinical context.

Several groups have reported that subjects with NSVTs over a wide range of cycle length (CL), are at higher adjusted risk of appropriate therapies [3, 5, 6] the risk being highest in those with a greater burden, defined as more than 5 episodes in the six months following the implant [4]. However, we do not have updated information on the relationship between the CL of NSVTs and the therapies subsequently applied by ICDs. This sub-analysis of our previously published experience [4] aims to determine the ability of CL of NSVT episodes (identified upon ICD interrogation during the first 6 months after implant) to predict the incidence and characteristics of tachycardias that will subsequently produce appropriate therapies.

## Methods

### Study population

Patients with left ventricular dysfunction (LVEF < 45%) and an ICD, implanted according to standard indications and without Cardiac Resynchronization Therapy, were studied [4]. Subjects were consecutively enrolled from January 2006 through to December 2013 and were followed up until June 2014. Vital status was available for 100% of the individuals at the end of the follow-up period, and patient recruitment was carried out at the time of ICD implantation.

The study complied with the Declaration of Helsinki. Enrolment of the patients followed once the protocol had been approved by the institutional review board, and informed consent was obtained from all patients.

### ICD programming

Detection and therapy programming were standardized and included three zones: ventricular fibrillation (VF): cycle length (CL) < 250 ms, ventricular tachycardia zone 1 (fast VT): CL from 250 to 320 ms, and ventricular tachycardia zone 2 (slow VT): CL from 321 to 400 ms.

In the case of the Medtronic devices, Ventricular Fibrillation detection required that 18 of the last 24 R–R intervals had a cycle length (CL) of < 250 ms (> 240 bpm). The fast VT detection zone was defined within the VF zone (fast VT zone via ventricular fibrillation). When any of the final 8 R–R intervals preceding the moment of detection was < 250 ms (> 240 bpm), the episode was classified as VF and received a high energy shock. When all of the last 8 R–R intervals were > 250 ms (< 240 bpm), the episode was detected as fast VT, and slow VT detection required 16 consecutive intervals.

For the Boston Scientific devices, the episodes were detected when 8 out of 10 RR intervals had a CL within the detection interval, and at least 6 out of every 10 RR intervals were within the predefined limits for VT detection, during the subsequent 2.5 s in fast and 5 s in slow VT.

Starting from 1 January 2013, the detection criteria were modified to 30/40 for fast VT and 40 consecutive RR intervals for slow VT (for the Medtronic devices), and to 8 (fast VT) and 16 s (slow VT) for the Boston Scientific devices.

The first ATP therapy in the fast VT zone was a single antitachycardia pacing (ATP) sequence (5-pulse-burst pacing train at 84% of the VT CL) and failed ATP was followed by a shock and then additional shocks, as necessary. Therapies for slow VT included 3 consecutive ATP bursts of 15 pulses at 91% of the VT CL, with no decrement. Failed ATP was followed by a sequence of shocks.

Additional criteria (onset, stability and morphology) were programmed in the slow VT zone. All devices were programmed to store the far-field electrogram before the onset of the detected episodes to aid in rhythm classification. In the Medtronic devices the “Smart mode” was programmed to “off”.

All tip-to-ring- and far-field-stored electrograms from spontaneous episodes were classified using predetermined criteria based on visual inspection and comparison with sinus rhythm electrograms [7, 8]. Classification of ventricular and supraventricular tachycardias was performed by two independent investigators, and a third investigator was consulted in cases where there was a lack of consensus.

### Definitions

NSVT was defined as any ventricular tachyarrhythmia with > 5 beats at ≥ 150 bpm terminating spontaneously before therapy that occurred within the first six months after ICD implant and which was not preceded by any arrhythmia causing appropriate therapy. Monomorphic VT (MVT) required a stable morphology of the electrogram, whereas in VF the morphology was changing beat to beat, with a CL < 250 ms.

### Statistical analyses

Statistical analysis was performed using the 25 version for Windows (SPSS Inc., Chicago, Illinois). Normal and continuous variables were described by mean and 95% confidence interval (CI) or standard deviation, whereas categorical variables were summarized by the number of patients and percentage. Comparison of the categorical variables was performed with the Chi-square test (or Fisher’s exact test if n < 5). Comparison of 2 normal variables (determined by the Kolgomorov–Smirnov test) and continuous variables was done using the Student’s t test. Comparison of > 2 continuous variables was performed using the ANOVA test.

To determine both the mean CL of NSVTs and MVT episodes, adjusted for multiple episodes per patient, the Generalized Estimating Equations Method (GEEM) was used in the calculations and comparisons [9].

The quantification of the linear relationship between two quantitative variables was studied by calculating the Pearson’s correlation coefficient (r Coefficient). The Bland–Antman method was used to evaluate the agreement between the values of two quantitative variables. Comparisons of cumulative incidences were carried out using the log-rank test. Univariate and multivariate Cox proportional hazards regression analysis was used to evaluate the contribution of baseline clinical factors [age, gender, diabetes, left ventricular ejection fraction, functional class (NYHA), etiology (ischemic vs. non-ischemic), indication (primary vs. secondary prevention), QRS duration (ms), atrial fibrillation, body mass index, serum creatinine (mg/dl), medical treatments (including amiodarone), burden of NSVTs and CL of NSVTs] to the occurrence of appropriate therapies, appropriate shocks, appropriate therapies due to MVT and appropriate therapies due to VF. Univariate variables with a *p* < 0.1 were included in a multivariate Cox-regression model. A *p* value < 0.05 was considered significant.

## Results

### Patients, follow-up and appropriate therapies

A total of 416 patients were consecutively studied. Their baseline characteristics are displayed in Table 1. They were followed for a mean of 41 ± 27 months from ICD implant.

**Table 1:** Baseline characteristics and outcomes of patients according to the episodes of nonsustained ventricular tachycardia

Variable	All patients	Patients without episodes	Patients with 1–5 episodes	Patients with > 5 episodes	*p* value
	n = 416	n = 166 (40)	n = 130 (31)	n = 120 (29)	
Age, years	65 ± 11	63 ± 12	65 ± 12	69 ± 9	< 0.001^†^
Male gender	87%	87%	88%	87%	0.9*
Ischemic etiology	62%	63%	62%	63%	0.8*
Secondary prevention	37%	35%	38%	37%	0.8*
Atrial fibrillation	22%	15%	20%	32%	0.002*
New York Heart Association Functional Class > 1	63%	56%	64%	71%	0.036*
Diabetes	25%	28%	22%	24%	0.5^†^
Left ventricular ejection fraction (%)	30 ± 8	30 ± 8	30 ± 9	30 ± 7	0.8^†^
QRS duration (ms)	118 ± 24	114 ± 23	120 ± 22	121 ± 26	0.018^‡^
Serum creatinine (mg/dl)	1.22 ± 0.48	1.15 ± 0.33	1.22 ± 0.37	1.32 ± 0.7	0.012^†^
Previous clinical nonsustained ventricular tachycardias	36%	11%	39%	51%	< 0.001*
Statins	62%	63%	61%	59%	0.7*
Beta-blockers	76%	83%	82%	70%	0.011^§^
Angiotensin Converting Enzyme Inhibitors or Angiotensin II Receptor Blockers	86%	86%	85%	89%	0.7*
Amiodarone	13%	8%	15%	20%	0.011*
Digoxin	18%	9%	22%	25%	0.001^||^
Appropriate therapy	46%	27%	48%	70%	< 0.001^¶^
Appropriate therapy due to MVT	44%	26%	45%	66%	< 0.001^¶^
Appropriate therapy due to VF	6.5%	3%	7%	12%	0.026^¶^
Appropriate shock	27%	16%	25%	44%	< 0.001^¶^
Cardiac mortality	17%	7%	18%	32%	< 0.001^¶^

Patients are classified according to the tertiles of NSVT episodes

*Chi-square test for trend; ^†^ANOVA for all comparisons; ^‡^first group versus others, ANOVA test; ^§^third tertile versus others, Chi-squared test; ^||^first group versus others, Chi-squared test; ^¶^Log-rank test for trend

**Fig. 1:**
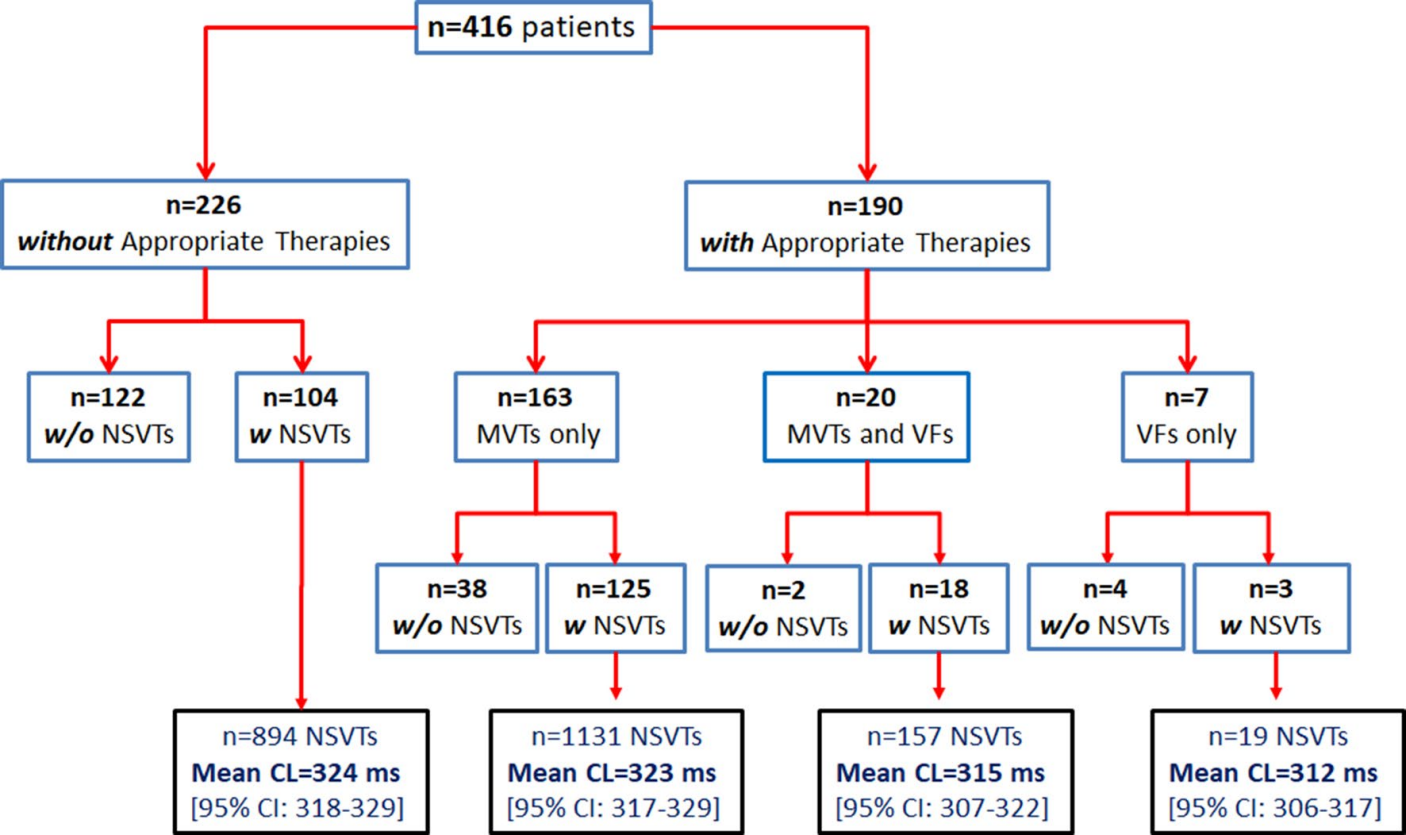
Diagram showing the relationship between the incidence of appropriate therapies (and their index arrhythmia) with the CL of preceding NSVT episodes

During follow-up, 1491 appropriate therapies (1441 due to monomorphic VT [MVT] and 50 due to VF) were recorded in 190 patients. Figure 1. Of the total of appropriate therapies, there were 439 shocks that occurred in 113 (27%) subjects.

### NSVT episodes occurring early

A total of 2335 episodes of NSVTs were documented during the first 6 months following ICD implant. After excluding 134 episodes that lacked stored electrograms, we analyzed 2201 NSVTs (10 + 7 beats), which occurred in 250 of the 416 patients. The median number of NSVTs per patient was 2 (IQR = 0–7). Patients were classified into three groups according to the tertiles of the NSVTs burden: zero (n = 166), 1–5 (n = 130) and > 5 (n = 120). Table 1.

### Cycle length of NSVT

The adjusted mean CL of NSVTs was 323 ms (95% CI 318–328). One hundred and eleven NSVTs had a CL ≤ 300 ms; they occurred in 27 patients.

As shown in Table 2, the adjusted mean CL of unsustained episodes was significantly lower in primary prevention patients and in those with > 5 episodes. In contrast, the CL of NSVT episodes was longer in subjects under beta-blocker treatment. We found no differences in the mean CL of NSVTs depending on the etiology, functional class or severity of the left ventricular dysfunction.

**Table 2:** Adjusted mean CL of NSVT episodes according to different variables

Variable	Mean adjusted CL of NSVT episodes^a^, ms	Statistical analysis
Ischemic etiology	323 (95% CI 319–327) versus 324 (95% CI 318–328)	95% CI of the difference (− 3; 5) * p* = 0.7
Primary prevention	320 (95% CI 318–323) versus 327 (95% CI 321–330)	95% CI of the difference (2; 10) * p* = 0.008
LVEF ≤ 35%	324 (95% CI 319–328) versus 320 (95% CI 316–327)	95% CI of the difference (− 4; 3) * p* = 0.2
Functional class > 1 (NYHA)	324 (95% CI 319–328) versus 322 (95% CI 317–326)	95% CI of the difference (− 7; 2) * p* = 0.2
Beta-blocker treatment	326 (95% CI 322–330) versus 316 (95% CI 309–321)	95% CI of the difference (5; 14) * p* < 0.001
> 5 NSVT episodes	321 (95% CI 317–325) versus 325 (95% CI 320–330)	95% CI of the difference (− 10; − 2) * p* = 0.004
≥ 1 appropriate therapies	322 (95% CI 317–328) versus 324 (95% CI 318–329)	95% CI of the difference (− 2; 3) * p* = 0.3
≥ 1 appropriate shocks	322 (95% CI 318–326) versus 325 (95% CI 320–329)	95% CI of the difference (− 1; 6) * p* = 0.2
≥ 1 appropriate therapies due to MVT	322 (95% CI 319–327) versus 324 (95% CI 319–328)	95% CI of the difference (− 3; 4) * p* = 0.3
≥ 1 appropriate therapies due to VF	314 (95% CI 308–321) versus 324 (95% CI 320–328)	95% CI of the difference (− 18; − 4) * p* = 0.002

*LVEF* left ventricular ejection fraction, *NYHA* New York Heart Association, *NSVT* non-sustained ventricular tachycardia, *MVT* MONOMORPHIC VENTRICULAR tachycardia, *vf* ventricular fibrillation

^a^Variable present versus variable absent

### Predictors of electrical therapies among subjects with NSVT

The mean CL of NSVTs was similar in patients with or without appropriate therapies and appropriate shocks (Table 2).

Secondary prevention (HR = 1.7; 95% CI 1.3–2.3; *p* < 0.001), burden of NSVT episodes (HR = 1.05; 95% CI 1.04–1.07; *p* < 0.001) and beta-blocker treatment (HR = 0.7; 95% CI 0.5–0.9; *p* = 0.04) were associated independently with the incidence of appropriate therapies. The same variables appeared as independent predictors of appropriate shocks: secondary prevention (HR = 2.2; 95% CI 1.6–3.2; *p* < 0.001), burden of NSVTs (HR = 1.04; 95% CI 1.02–1.06; *p* < 0.001) and beta-blocker treatment (HR = 0.6; 95% CI 0.4–0.9; *p* = 0.04). However, the CL of NSVTs was not a predictor of either appropriate interventions (HR = 0.4; 95% CI = 0.4–1.5; *p* = 0.6) or appropriate shocks (HR = 0.5; 95% CI = 0.3–1.6; *p* = 0.4).

As shown in Table 3, the burden of NSVTs per patient maintained its independent predictive capacity on the incidence of appropriate therapies over the whole spectrum of CL values.

**Table 3:** Adjusted hazard ratio of NSVT burden according to the mean CL of the episodes (adjusted for multiples episodes per patients, GEEM)

Mean CL of NSVTs (ms)	Number of patients	HR (95% CI)	*p* value
≤ 310	58	1.06 (1.01–1.12)	0.03
311–330	156	1.05 (1.03–1.08)	< 0.001
331–400	36	1.07 (1.02–1.13)	0.012

Multivariate Cox proportional hazards regression analysis

*NSVT* nonsustained ventricular tachycardia, *CL* cycle length, *GEEM* generalized estimating equations method, *HR* hazard ratio, *CI* confidence interval

### Appropriate therapies due to monomorphic VT

The 1441 MVT occurred in 183 patients. Figure 1. The mean CL of MVT was 335 ms (95% CI interval 324–340). There were 446 (31%) fast VT episodes with a CL ≤ 320 ms that occurred in 97 (23%) individuals. As shown in Table 2, the mean CL of NSVTs was similar in patients with or without appropriate therapies due to MVT. By multivariate analysis (Cox-regression), the indication for secondary prevention (HR = 1.6; 95% CI = 1.2–2; *p* = 0.4) and a NSVT burden > 5 episodes (HR = 2; 95% CI = 1.6–2.; *p* = 0.002) appeared to be independent predictors of appropriate therapies due to MVT. However, neither the beta-blocker treatment (HR = 0.7; 95% CI = 0.5–1.06; *p* = 0.1) nor the mean CL of the NSVT episodes (HR = 0.5; 95% CI = 0.4–1.5; *p* = 0.5) reached statistical significance.

**Fig. 2:**
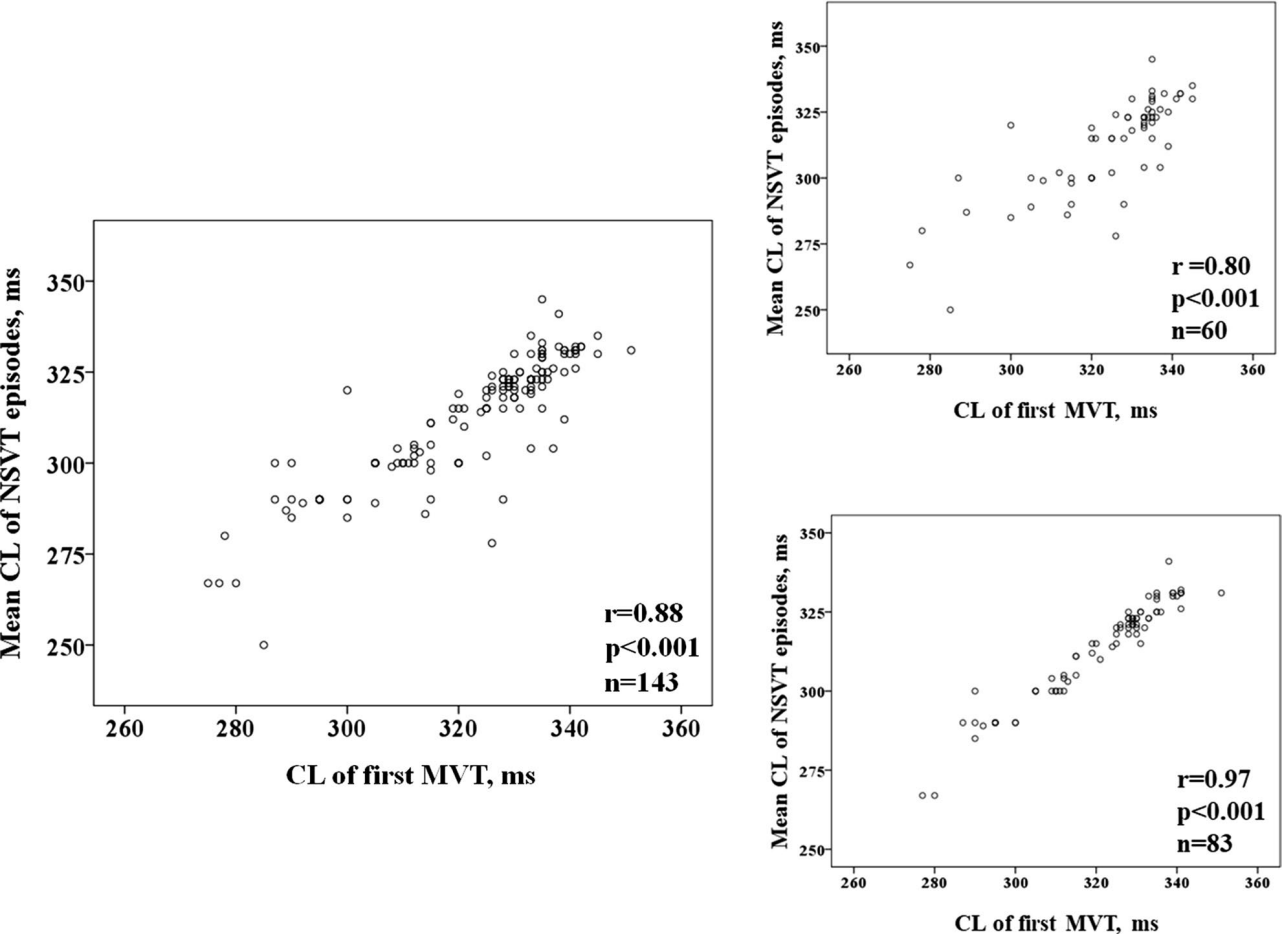
Correlation between the mean CL of NSVT episodes and the CL of the first MVT leading to appropriate therapy. Left: all patients (n = 143). Right: Top individuals with 1–5 NSVT episodes (n = 60). Bottom subjects with > 5 NSVT (n = 83)

Among patients who presented NSVT and MVT episodes (n = 143), we analyzed the relationship between the adjusted mean CL of the NSVTs (n = 1288 episodes) and the CL of the first appropriate therapy due to MVT occurring subsequently. Although the mean CL was shorter in NSVT [324 ms (95% CI 320–227)] compared to MVT episodes [330 ms (95% CI 326–334)], we found a significant correlation between the two (Fig. 1, left), with similar robustness in individuals with ischemic (r coefficient = 0.86; n = 90) versus non-ischemic cardiomyopathy (r = 0.90; n = 543, and in primary (r = 0.86; n = 73) versus secondary prevention (r = 0.90; n = 70). The strongest correlation was observed in subjects with > 5 NSVTs, as shown in Fig. 2.

**Fig. 3:**
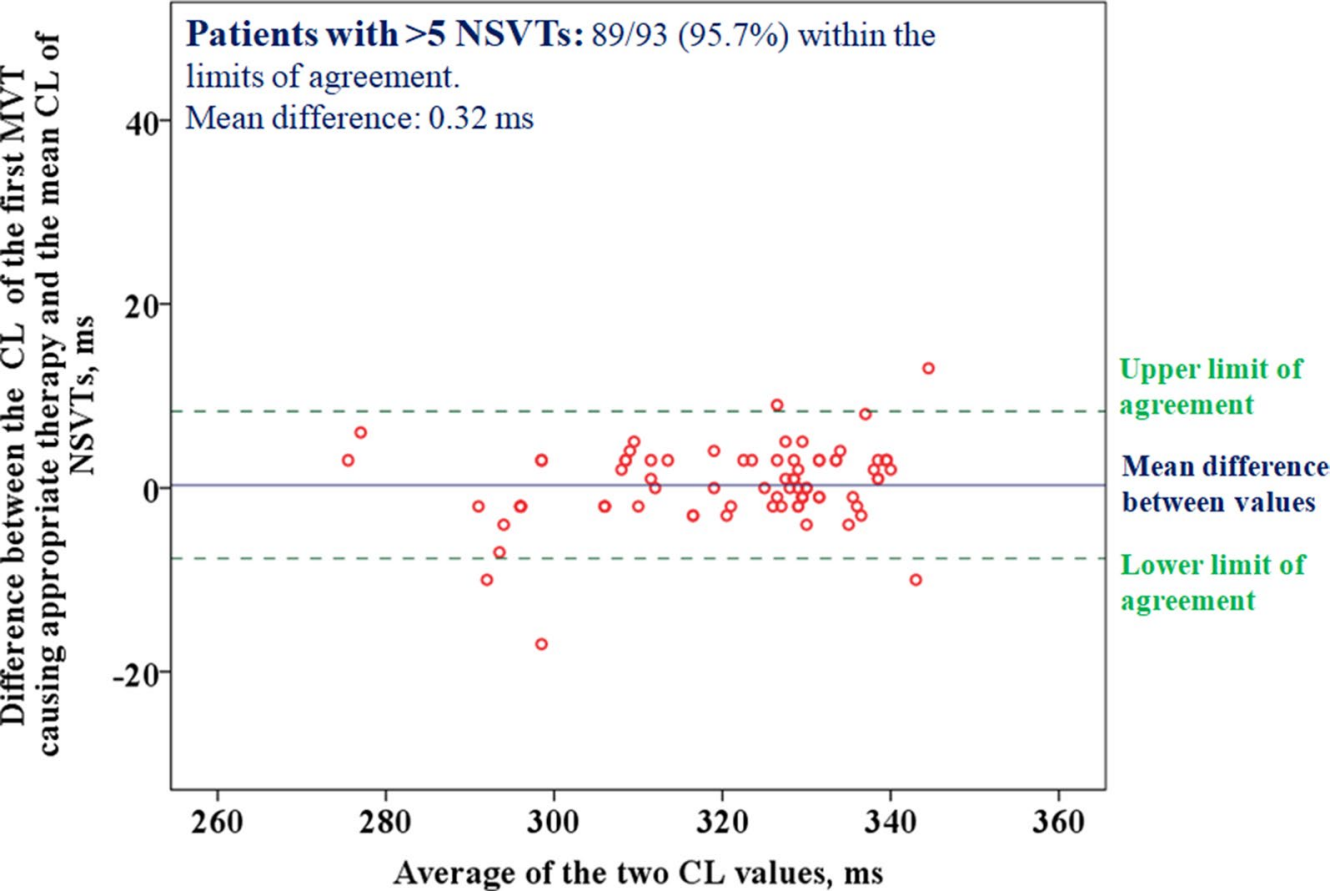
Bland–Altman graph assessing the agreement between the mean CL of NSVT episodes adjusted for multiples episodes per patient (GEEM) and the CL of the first MVT that resulted in appropriate therapy among patients with > 5 NSVTs. Each red circle represents a patient. Therefore, in these subjects the CL of the previous non-sustained episodes and the CL of the first subsequent MVT is virtually the same

We also analyzed the agreement between both values. The difference between the two values was 2 ± 8.3 ms, with only 7.6% (11/145) of patients in whom the difference between the two CL was outside the concordance limits. The agreement was greater, again, in individuals with > 5 NSVTs. As shown in Fig. 3, in more than 95% of such patients both values were within the interval of agreement (0.32 ± 4 ms).

### Appropriate therapies due to VF

Fifty episodes of VF occurred during the follow-up in 27 (6.5%) patients; of these, 21 presented at least one NSVT. Figure 1. As shown in Table 1, the cumulative incidence of VF increased according to the NSVTs burden.

**Fig. 4:**
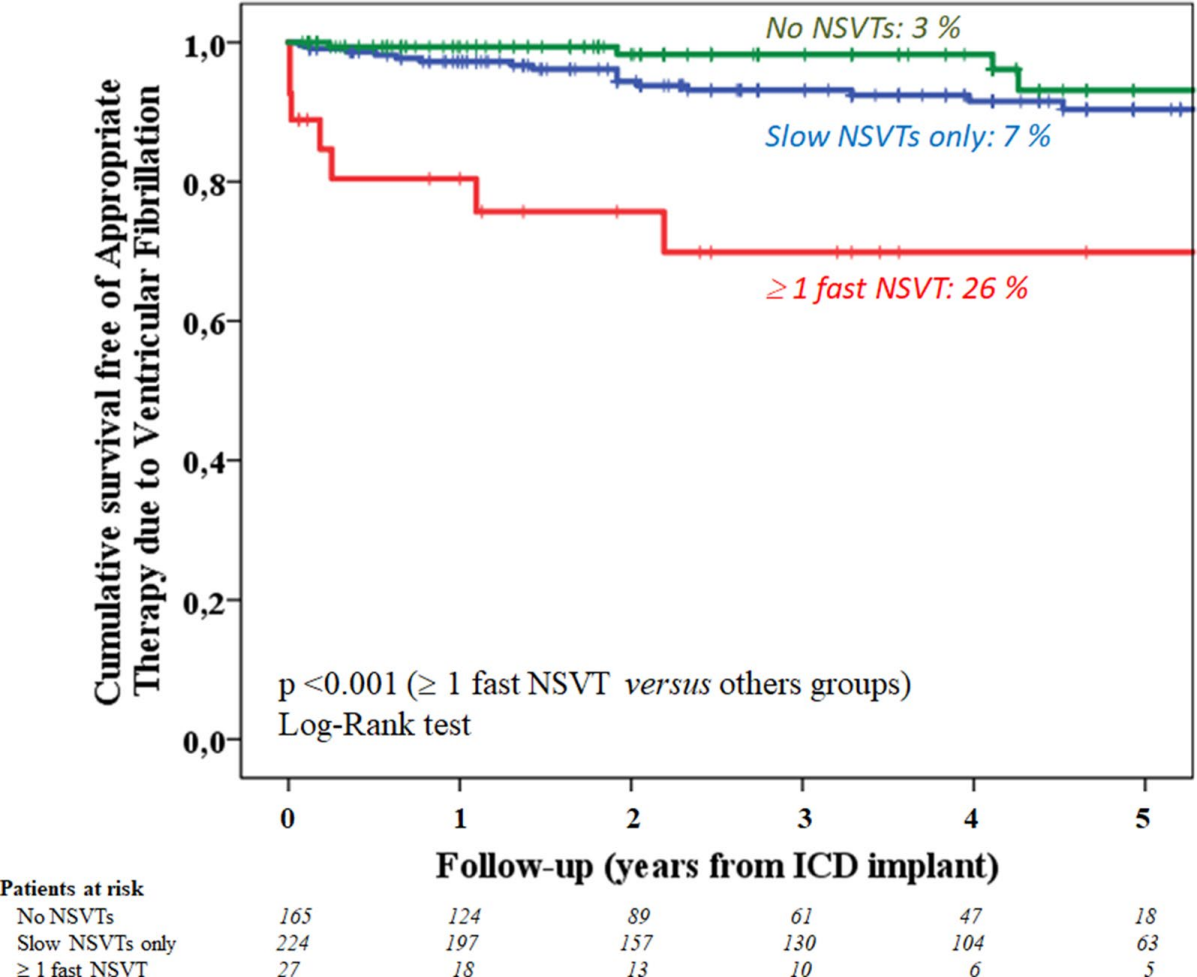
Kaplan–Meier estimates of the probability of appropriate therapies due to VF according to the previous incidence of fast NSVT episodes. Patients with at least one episode of NSVT with CL ≤ 300 ms (n = 27) had a significantly higher subsequent incidence of appropriate therapies due to VF than subjects without NSVTs (n = 165) or with only slow NSVT episodes (n = 224)

The adjusted mean CL of the NSVTs was significantly lower in subjects with episodes of VF. Table 2. By multivariate analysis, the adjusted mean CL of NSVT episodes was the only independent predictor of appropriate therapies due to VF: HR = 0.97 (95% CI 0.95–0.99); *p* < 0.005. Neither the burden of NSVTs > 5 episodes (HR = 1.6; 95% CI 0.6–4.2; *p* = 0.3) nor the beta-blocker treatment (HR = 0.5; 95% CI 0.2–1.1; *p* = 0.1), nor the indication for secondary prevention (HR = 1.6; 95% CI 0.7–3.6; *p* = 0.3) reached statistical significance. As shown in Fig. 4, patients with at least one fast episode of NSVT (CL ≤ 300 ms) experienced a higher incidence of appropriate therapies due to VF.

## Discussion

### Main findings

In this work we have analyzed the relationship between the CL of NSVTs episodes occurring early (i.e., in the first six months after implantation of an ICD) and the arrhythmias underlying the therapies applied by the devices. The main findings of this study are: (1) unlike the burden, the CL of NSVT episodes occurring early after an ICD implantation is not an independent predictor of appropriate therapies; (2) the CL of the first MVT causing appropriate therapy is closely related to the adjusted mean CL of precedent NSVT episodes and such relationship (expressed as a significant correlation and concordance) is especially robust in patients with more than 5 NSVTs; and (3) patients who present with fast episodes of NSVTs are at significantly increased risk of appropriate therapies due to VF.

### NSVT episodes and electrical therapies

The occurrence of episodes of NSVT in subjects with left ventricular dysfunction is associated with an increase risk in arrhythmic events. This is true both for NSVTs observed during continuous ambulatory ECG monitoring [10] and for their documentation during ICDs interrogation [3]. Although there are few updated data on myocardial tissue characterization, it has classically been assumed that, in subjects with ischemic heart disease, the electrophysiological substrate of subjects with only episodes of NSVT, sustained MVT or VF is different. Therefore, both the presence of pathological electrograms is more frequent and the endocardial activation time of the left ventricle is longer in individuals with MVT [11]. In addition, regardless of etiology, it is assumed that reentry is the mechanism underlying the vast majority of MVTs among subjects with scars and ventricular dysfunction [12].

The arrhythmogenic substrate in subjects with myocardial scars is not stable, but progresses over time [13], which may justify the clinical evolution from nonsustained to sustained forms of VT [14]. In this line, Elin et al*.* have shown the existence of a significant association between the induction of NSVTs after catheter ablation and the recurrence of sustained MVT [15]. In addition, different burdens of unsustained tachycardias may correspond to different degrees of substrate evolution.

### CL of NSVT episodes and ICD interventions

Several studies have found an association between the incidence of NSVTs and the occurrence of appropriate therapies. The CL of the non-sustained episodes analyzed in such series has been variable: from evaluating only rapid episodes (CL < 320 ms) [3] to analyzing NSVTs with CL < 400 ms [4, 6]. However, in none of them has been studied the impact of CL on the incidence and characteristics of arrhythmias that subsequently produce appropriate therapies. According to our data, unlike the burden, the CL of NSVTs is not a predictor of the subsequent incidence of appropriate therapies. In other words, the burden of NSVTs per patient maintained its independent predictive capacity on the incidence of appropriate therapies over the whole spectrum of mean CL values (Table 3).

Among ICD patients with left ventricular dysfunction, the vast majority of appropriate therapies are caused by MVT. The mean CL of NSVTs is slightly lower than that of the MVT which causes the first appropriate therapy, surely because, among ICD patients, the vast majority of MVTs slow discreetly from detection to therapy [16]. Remarkably, after analyzing the mean CL of the NSVTs and that of the first MVT, which subsequently led to an appropriate therapy, we observed a significant correlation and agreement between the two that was especially robust in subjects with more unsustained episodes. It could be thought that it was the same tachycardia with different duration, a circumstance that is impossible to confirm with our data. In any case, these data indicate that the arrhythmic substrate underlying such individuals is not only more active, but also more specific. In this way, the CL of the NSVTs could be used as a reference for the programming of the detection windows.

### VF episodes

In patients with ICD and left ventricular systolic dysfunction appropriate therapies due to VF are rare, accounting for less than 20% of events and affecting a minority of subjects [17,18]. Their incidence appears to be similar regardless of etiology (ischemic vs. non-ischemic [19]) and indication (primary vs. secondary prevention [18]). Only individuals with a previous history of VF appear to be more at risk [20].

According to our data, the CL of the NSVTs is a predictor of subsequent appropriate therapies due to VF. In our series, 6.5% of patients had at least one rapid episode of NSVT (CL ≤ 300 ms): one out of such subjects will undergo appropriate therapies for VF in the medium and long term. These data should be taken into account when programming the devices in selected individuals, to avoid delaying detection in the VF zone, since VF episodes produce a rapid hemodynamic impact, including loss of consciousness if not treated quickly.

## Limitations

The detection algorithms used until 2013, although validated in several multicenter studies, may have been too sensitive, so that the incidence of appropriate therapies may be overestimated.

We do not have data on the compared morphology (i.e., far-field electrograms) of the NSVTs versus the subsequent MVT that resulted in appropriate therapy. Although this analysis could have provided additional information regarding their anatomical origin, it is often not possible to carry it out because of the change in the morphology of the first beats of the VTs.

Finally, since we only analyzed patients with a single- or dual-chamber ICD, our findings are not applicable to cardiac resynchronization patients.

## Conclusions

Unlike the burden, the CL of NSVTs occurring early after an ICD implant, is not a predictor of having appropriate therapies in general, nor of appropriate therapies due to MVT. However, patients presenting with fast NSVT are at significant risk of appropriate therapies due to VF. In addition, all nonsustained events with a CL within the range explored in this study have prognostic impact.

There is some specificity between the mean CL of such NSVT episodes and that of the ventricular arrhythmias that subsequently lead to appropriate therapies, which may be useful when programming the devices.

Future efforts will need to be directed to determine which patients may be candidates for either antiarrhythmic drug therapy or catheter ablation for the elimination NSVT episodes and to assess whether arrhythmia suppression can improve outcomes in these patients.

## Data Availability

All material in the article is the property of the authors; no third-party permission is required for publication. The datasets used and/or analyzed during the current study are available from the corresponding author on reasonable request.

## Abbreviations

NSVTs: Nonsustained ventricular tachycardias
ICD: Impantable cardioverter-defibrillator
CL: Cycle length
VT: Ventricular tachycardia
VF: Ventricular fibrillation
MVT: Monomorphic ventricular tachycardia
LVEF: Left ventricular ejection fraction
ATP: Antitachycardia pacing
GEEM: Generalized estimating equations method
HR: Hazard ratio
OR: Odds ratio
